# Supplementary material for: Drebrin-mediated microtubule–actomyosin coupling steers cerebellar granule neuron nucleokinesis and migration pathway selection
Source: Nat Commun. 2017 Feb 23;8:14484. doi: 10.1038/ncomms14484 (PMC5331215; doi:10.1038/ncomms14484)
Supplement: Supplementary Information — Supplementary Figures, Supplementary Note, and Supplementary References [file ncomms14484-s1.pdf]

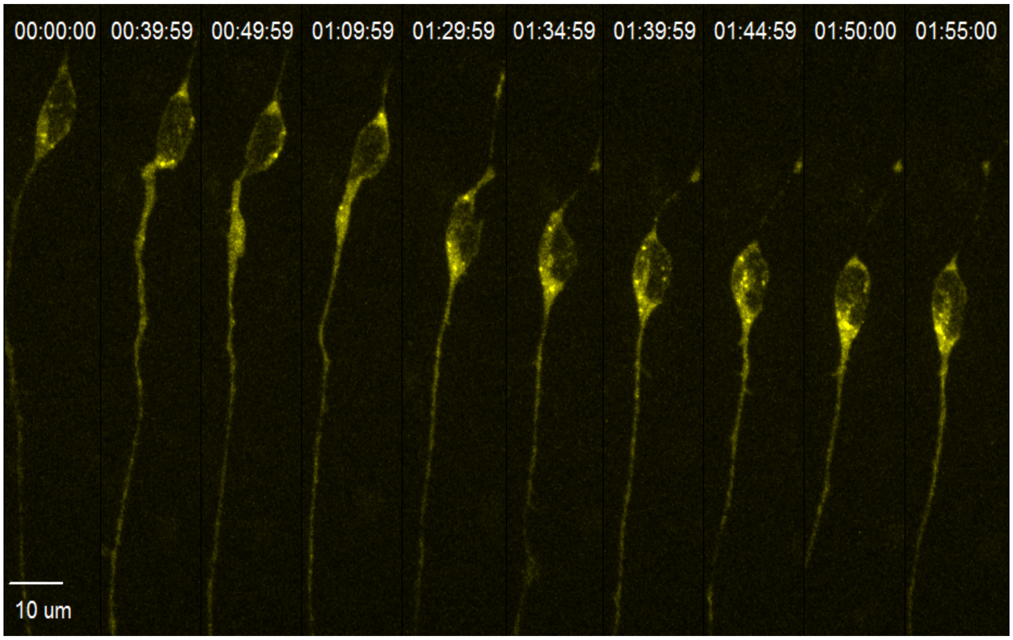

Supplementary Figure 1. Time-lapse imaging of cytoplasmic dynein in a CGN migrating *in vitro*. CGNs were transfected with expression vectors encoding 2xVenus Cytoplasmic Dynein. Time-lapse imaging was used to monitor two-stroke nucleokinesis. Dynein foci are located near the nucleus and accumulate in the cytoplasmic dilation of the proximal leading process prior to somal translocation. Scale bar equals ten microns.

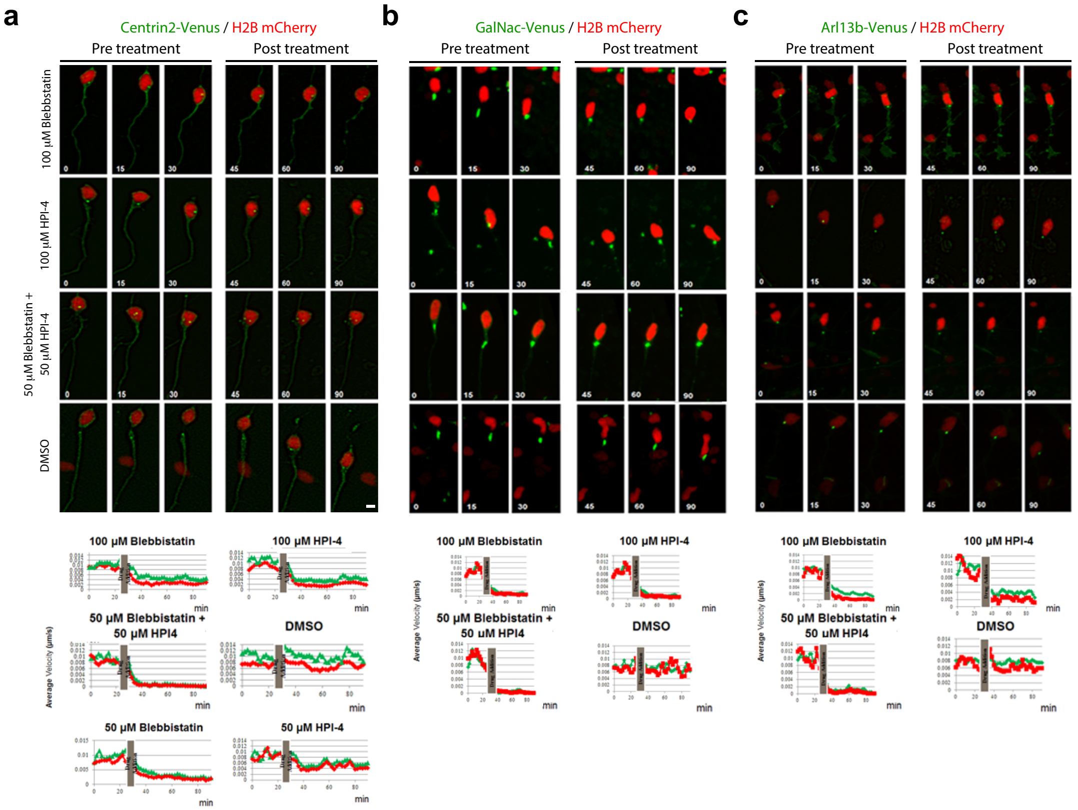

Trivedi et al. Supplementary Figure 2

Supplementary Figure 2. Myosin II and cytoplasmic dynein motor activity are required for both phases of the two-stroke motility cycle. CGNs were transfected with expression vectors encoding Centrin2-Venus (**a**), GalNAcT2-YFP (**b**), or Arl13b-Venus (**c**) in combination with H2B-mCherry. Time-lapse imaging was used to monitor two-stroke nucleokinesis in migrating CGNs. After the cells were allowed to migrate for approximately 20 min, the indicated amounts of blebbistatin or HPI-4 were added to the culture and imaging continued for a further hour. The quantitation curves below the time-lapse images show average velocities before and after adding the drug; the cumulative statistics are reported in the Results section. Adding either drug potently inhibited forward movement of organelles and nuclei. Scale bar equals five microns.

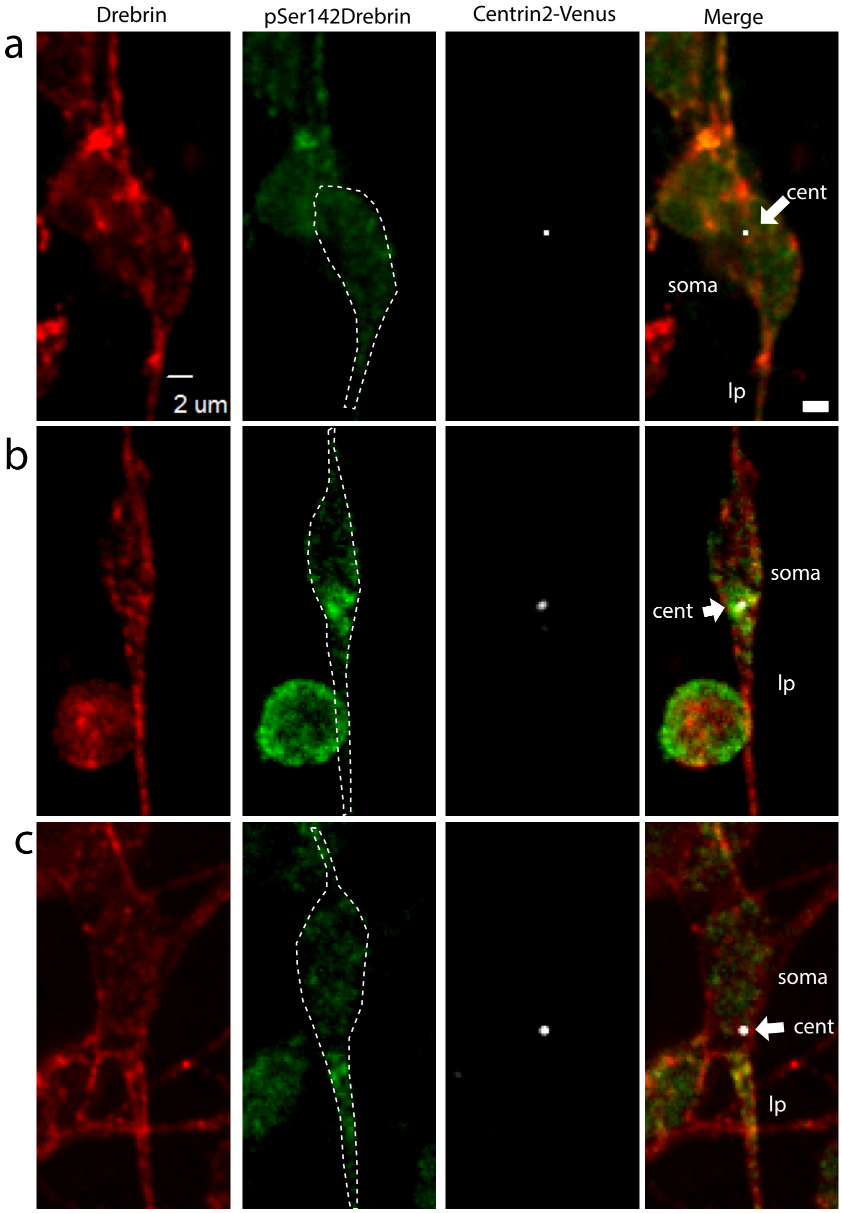

Supplementary Figure 3. Alteration of drebrin Ser142 phosphorylation during the two-stroke migratory cycle. CGNs were transfected with expression vectors encoding centrin2-Venus, fixed after 24 hours of culture and stained with antibodies recognizing drebrin and drebrin phosphorylated on Ser142. (a) In first stage of the migratory cycle where the centrosome is not yet polarized Ser142 phosphorylated drebrin is diffusely localized throughout the neuronal cell body. (b) At the initial stages of centrosome polarization, Ser142 phosphorylated drebrin appears to be polarized to the portion of the soma where the centrosome is located. (c) In a cell with a drebrin collar and dilated leading process, a significant fraction of Ser142 phosphorylated drebrin is located in the drebrin collar and dilation. Scale bar equals two microns.

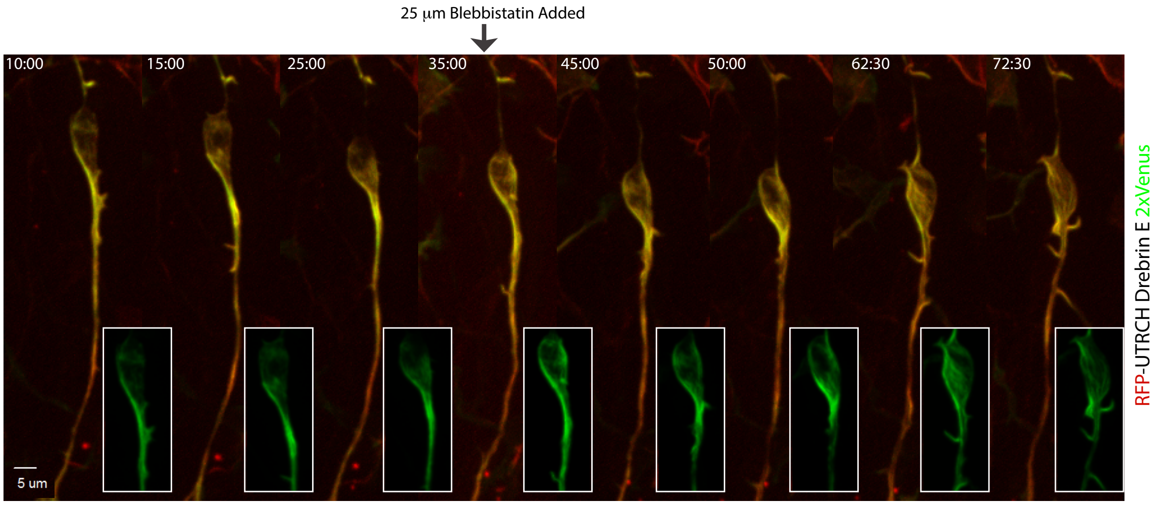

Supplementary Figure 4. Myosin II motor activity is required for drebrin dynamics in migrating neurons. CGNs were transfected with expression vectors encoding drebrin E-2x Venus (green) or RFP-UTRCH-ABD (f-actin label, red). Time-lapse imaging was used to monitor drebrin and actin dynamics in migrating cells. After 35 minutes of pretreatment migration, 25 µM blebbistatin was added and imaging continued for a further 40 minutes. Blebbistatin treatment potently inhibited migration and the leading process drebrin flow. Inset shows a enlarged view of the drebrin 2xVenus signal in the proximal leading process and soma. Scale bar=5 µm.

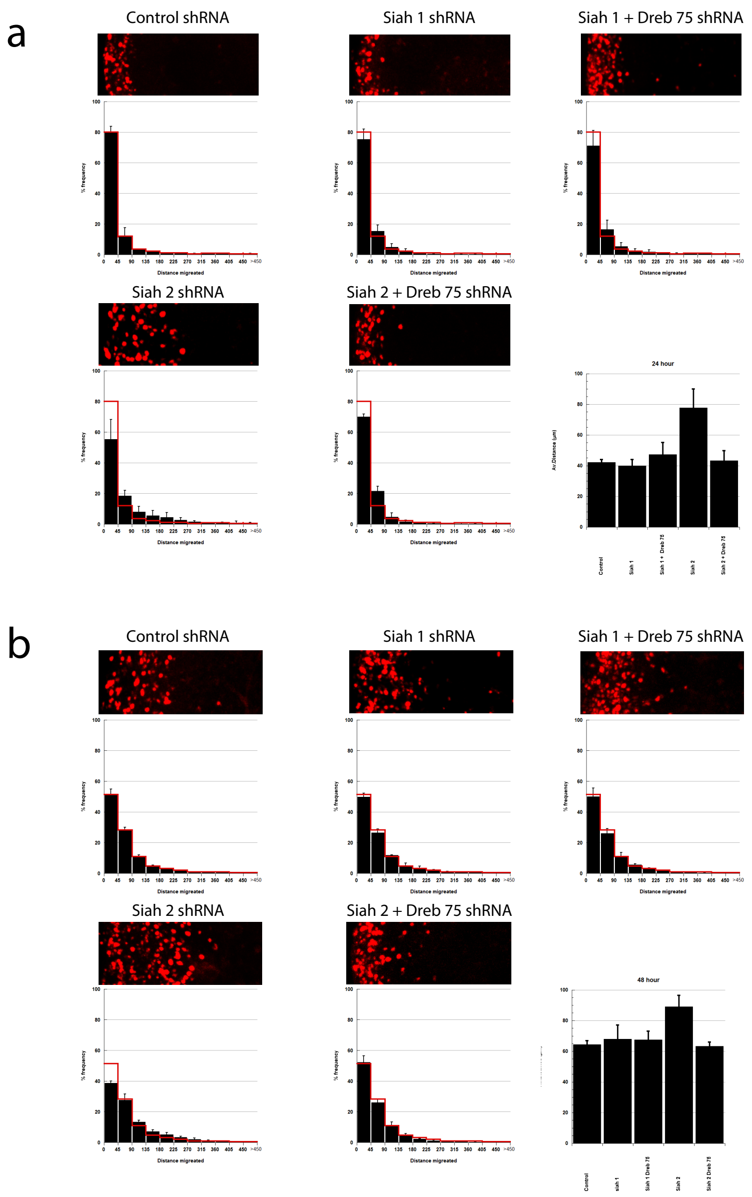

Supplementary Figure 5. Epistasis analysis of drebrin and Siah function in *ex vivo* cerebellar slices. Cerebella from P7 mice were dissected, electroporated, sectioned, and grown in *ex vivo* culture for (**a**) 24 h or (**b**) 48 h. The cells were electroporated with a vector encoding H2B-cherry (red nuclear stain) in combination with the indicated expression vectors (*control* shRNA, *Siah1* shRNA, *Siah2* shRNA, or *drebrin* 75 shRNA). Each representative image is oriented with the cerebellar slice surface to the left; the red nuclei in the center or right of the image indicate cells that have left the GZ. The histograms below each representative image show the binned migration distance distribution for each condition (n≥5351 cells analysed for each condition). The graphs to the lower right show average migration distances. *Siah2* silencing shows the expected precocious GZ exit at 24 h and enhanced migration at 48 h ($P<0.05$ by Student's *t*-test), both of which are reduced by *drebrin* silencing. Note that in the presence of *Siah1* silencing, *drebrin* loss of function does not inhibit migration as we observed in Figure 5, suggesting that Siah1 alters enough drebrin expression to render drebrin silencing ineffective.

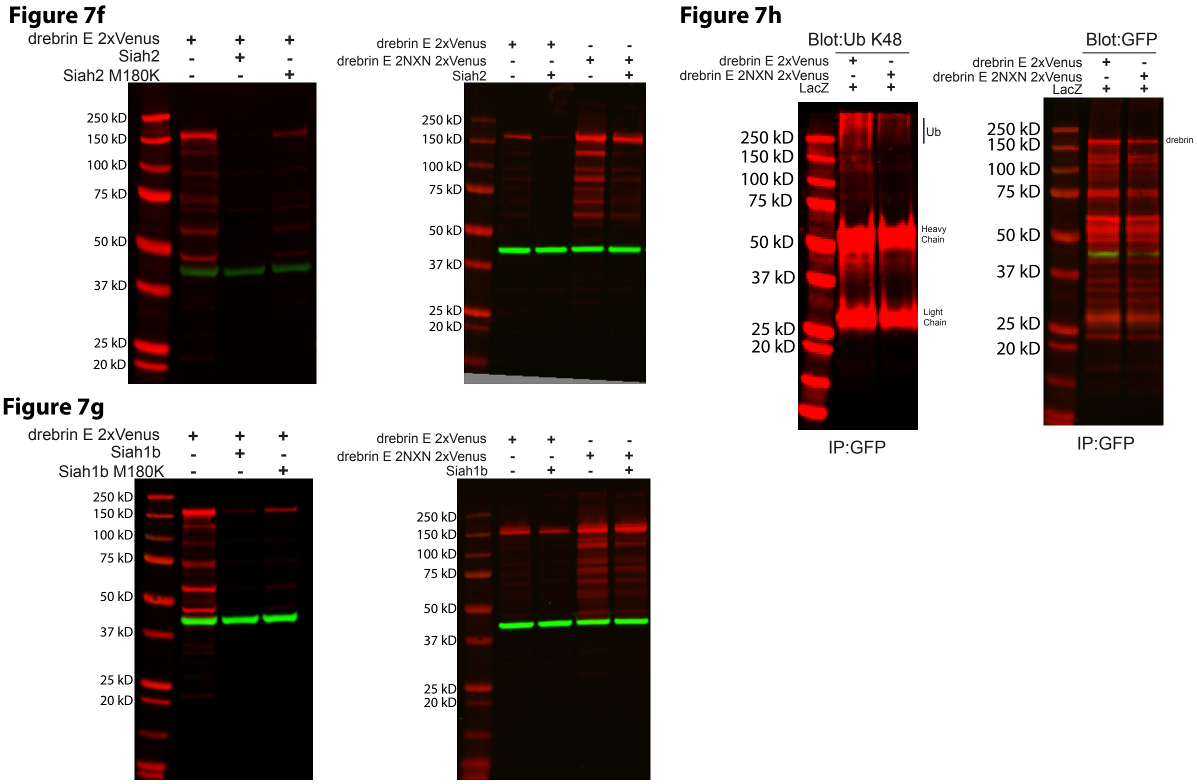

Supplementary Figure 6 . Uncropped western blots from Figure 7f, g and h.
Molecular weight marker and drebrin E 2x Venus, drebrin E 2xNxn 2xVenus, or K48 Ubiquitin signals are in red. Actin loading control is in green. See Figure 7 and legend for experimental details.

Supplementary Note 1

Dynein and myosin II contribute similarly to the CGN two-stroke motility cycle.

The two-stroke nucleokinesis cycle has been the standard model for dissecting the cytoskeletal components required for organelle and nuclear positioning events during neuronal migration[1-6]. Before assessing potential microtubule-actomyosin interactions, we clarified the role of the microtubule and actin motors in CGNs, given that their relative contributions and localizations are controversial in other neuronal populations described in the literature. We previously established that f-actin and myosin II motors act in the proximal leading process during CGN migration and that microtubule structures, such as Map2C-labeled microtubules and the centrosome, enter the proximal leading process before somal translocation[7, 8]. We now assessed cytoplasmic dynein localization in CGNs with a fluorescent reporter constructed by fusing a tandem repeat of Venus to the N-terminus of the full-length mouse cytoplasmic dynein1 cDNA. Time-lapse imaging of CGNs nucleofected with an expression vector encoding 2xVenus-cytoplasmic dynein revealed that dynein localizes diffusely in the soma of neurons before migration (Supplementary Fig. 1). It then accumulates in the proximal leading process before somal translocation, which is consistent with the proposed role of dynein in the “cytoplasmic dilation”[9].

We next compared the relative contributions of actin-based (myosin II) and microtubule-based (cytoplasmic dynein) motor systems to two-stroke nucleokinesis. No previous study has included such a direct comparison or

investigated the role of each motor system in positioning multiple cytoplasmic organelles in the CGN system. We nucleofected CGNs with expression vectors encoding H2B-mCherry/Centrin2-Venus (nucleus/centrosome label), H2B-mCherry/GalNAcT2-YFP (nucleus/Golgi apparatus label) or H2B-mCherry/Arl13b-Venus (nucleus/primary cilia label) and used time-lapse imaging to assay the centrosome, Golgi apparatus, primary cilium, and nuclear movement before and after adding 100 μM blebbistatin, which inhibits myosin II motor activity, or 100 μM HPI-4, a novel cell-permeable cytoplasmic dynein inhibitor (Supplementary Fig. 2a–c). Blebbistatin reduced the mean nuclear velocity from 0.009±0.0001 (SE) μm/s to 0.003±0.0004 μm/s (n=46), the mean centrosome velocity from 0.01 ± 0.001 μm/s to 0.004±0.00007 μm/s (n=46), the mean Golgi apparatus velocity from 0.009±0.002 μm/s to 0.001±0.0003 μm/s (n=26), and the mean primary cilium velocity from 0.009±0.0007 μm/s to 0.001±0.00002 μm/s (n=50). Dynein inhibition phenocopied myosin II motor perturbation, as HPI-4 application reduced the mean nuclear velocity from 0.009±0.0008 (SE) μm/s to 0.002±0.0008 μm/s (n=55), the mean centrosome velocity from 0.01±0.0001 μm/s to 0.002±0.00008 μm/s (n=55), the mean Golgi apparatus velocity from 0.01±0.001 μm/s to 0.002±0.0007 μm/s (n=20), and the mean primary cilia velocity from 0.011±0.001 μm/s to 0.003±0.0006 μm/s (n=19) ($P<0.01$ by Student's *t*-test for all conditions). Surprisingly, when both blebbistatin and HPI-4 were applied to inhibit both motor systems simultaneously, these organelle velocities were further reduced compared to those under single-drug conditions. These results show that dynein motors accumulate dynamically in the proximal

leading process of migrating CGNs, which is also the location of actomyosin flow during the first phase of the two-stroke motility cycle. Whereas inhibiting myosin II or cytoplasmic dynein results in identical migration phenotypes, their simultaneous inhibition halts migration completely.

Supplementary References

1. Heng, J.I., A. Chariot, and L. Nguyen, *Molecular layers underlying cytoskeletal remodelling during cortical development.* Trends Neurosci, 2010. **33**(1): p. 38-47.
2. Govek, E.E., M.E. Hatten, and L. Van Aelst, *The role of Rho GTPase proteins in CNS neuronal migration.* Developmental neurobiology, 2011. **71**(6): p. 528-53.
3. Trivedi, N. and D.J. Solecki, *Neuronal migration illuminated: a look under the hood of the living neuron.* Cell Adh Migr, 2011. **5**(1): p. 42-7.
4. Evsyukova, I., C. Plestant, and E.S. Anton, *Integrative mechanisms of oriented neuronal migration in the developing brain.* Annu Rev Cell Dev Biol, 2013. **29**: p. 299-353.
5. Cooper, J.A., *Cell biology in neuroscience: mechanisms of cell migration in the nervous system.* J Cell Biol, 2013. **202**(5): p. 725-34.
6. Kawauchi, T., *Cellullar insights into cerebral cortical development: focusing on the locomotion mode of neuronal migration.* Front Cell Neurosci, 2015. **9**: p. 394.
7. Solecki, D.J., et al., *Myosin II motors and F-actin dynamics drive the coordinated movement of the centrosome and soma during CNS glial-guided neuronal migration.* Neuron, 2009. **63**(1): p. 63-80.
8. Trivedi, N., et al., *Leading-process actomyosin coordinates organelle positioning and adhesion receptor dynamics in radially migrating cerebellar granule neurons.* Neural Dev, 2014. **9**: p. 26.
9. Tsai, J.W., K.H. Bremner, and R.B. Vallee, *Dual subcellular roles for LIS1 and dynein in radial neuronal migration in live brain tissue.* Nat Neurosci, 2007. **10**(8): p. 970-9.
